# The Combined Use of *in Silico, in Vitro*, and *in Vivo* Analyses to Assess Anti-cancerous Potential of a Bioactive Compound from Cyanobacterium *Nostoc* sp. MGL001

**DOI:** 10.3389/fphar.2017.00873

**Published:** 2017-11-27

**Authors:** Niveshika, Ekta Verma, Shashank K. Maurya, Rajnikant Mishra, Arun K. Mishra

**Affiliations:** ^1^Laboratory of Microbial Genetics, Department of Botany, Banaras Hindu University, Varanasi, India; ^2^Biochemistry and Molecular Biology Laboratory, Department of Zoology, Banaras Hindu University, Varanasi, India

**Keywords:** EMTAHDCA, anticancer drug potential, *in silico*, *in vivo*, *in vitro* analysis

## Abstract

Escalating incidences of cancer, especially in developed and developing countries, demand evaluation of potential unexplored natural drug resources. Here, anticancer potential of 9-Ethyliminomethyl-12-(morpholin-4-ylmethoxy)-5,8,13,16-tetraaza -hexacene-2,3-dicarboxylic acid (EMTAHDCA) isolated from fresh water cyanobacterium *Nostoc* sp. MGL001 was screened through *in silico, in vitro*, and *in vivo* studies. For *in silico* analysis, EMTAHDCA was selected as ligand and 11 cancer related proteins (Protein Data Bank ID: 1BIX, 1NOW, 1TE6, 2RCW, 2UVL, 2VCJ, 3CRY, 3HQU, 3NMQ, 5P21, and 4B7P) which are common targets of various anticancer drugs were selected as receptors. The results obtained from *in silico* analysis showed that EMTAHDCA has strong binding affinity for all the 11 target protein receptors. The ability of EMTAHDCA to bind active sites of cancer protein targets indicated that it is functionally similar to commercially available anticancer drugs. For assessing cellular metabolic activities, *in vitro* studies were performed by using calorimetric assay *viz*. 3-(4,5-dimethylthiazol-2-yl)-2,5 diphenyltetrazolium bromide (MTT). Results showed that EMTAHDCA induced significant cytotoxic response against Dalton's lymphoma ascites (DLA) cells in a dose and time dependent manner with an inhibitory concentration (IC_50_) value of 372.4 ng/mL after 24 h of incubation. However, in case of normal bone marrow cells, the EMTAHDCA did not induce cytotoxicity as the IC_50_ value was not obtained even with higher dose of 1,000 ng/mL EMTAHDCA. Further, *in vivo* studies revealed that the median life span/survival days of tumor bearing mice treated with EMTAHDCA increased significantly with a fold change of ~1.9 and 1.81 corresponding to doses of 5 and 10 mg/kg body weight (B.W.) of EMTAHDCA respectively, as compared to the DL group. Our results suggest that 5 mg/kg B.W. is effective since the dose of 10 mg/kg B.W. did not show any significant difference as compared to 5 mg/kg B.W. Taken together, our findings based on *in silico, in vitro*, and *in vivo* analyses suggest that EMTAHDCA has potential anticancer effects, and thus, can be considered for cancer treatment.

## Introduction

Cancer is one of the world's deadliest diseases and is becoming a matter of worldwide concern. According to the World Health Organization (WHO), 7.9 million deaths (around 13% of all deaths) have been accounted in 2007 mainly due to the cancer; of which, 38% cancer incidences have been observed in developed countries, and 68% cases have been notified in developing countries (http://www.who.int/cancer/en/). A project of the International Agency for Research on Cancer (IARC), known as GLOBOCAN 2012, has estimated that more than 13.2 million deaths and nearly 21.4 million new cancer incidences will appear by 2030 (Bray et al., 2013; Ferlay et al., 2013). Escalating cancer evidences, ineffectiveness of anticancer drugs, and adverse side-effects of drug over-dose demand more investigations on identifying newer, broad-spectrum anticancer molecules from less explored, potential natural products. Development of modern technologies offers unique opportunities for finding and discovering new drug compounds from natural resources (Cragg et al., 1997). Over the past few years, natural products and their derivatives have been recognized as important sources of new medicines and therapeutic agents (Cragg and Newman, 2013). da Rocha et al. (2001) have mentioned about the importance of natural products derived from microbes in anticancer therapy. Various microorganisms *viz*. actinomycetes, fungi, and bacteria including cyanobacteria are known for producing bioactive metabolites (Bérdy, 2005; El-Elimat et al., 2012). Of these organisms, cyanobacterial cultivation without organic substrates is considered as a cost effective approach for scientific studies as compared to other microorganisms (Bullerjahn and Post, 2014; Dias et al., 2015).

Cyanobacteria are the most diverse group of oxygenic photosynthetic prokaryotic organisms distributed across a broad range of ecosystem (Singh et al., 2005; Sivonen and Börner, 2008). Nowadays, cyanobacteria have become a potential microorganism for biotechnological applications for producing wide array of unprecedented biologically active natural products with anticancer, antifungal, antimicrobial, antiviral, antioxidants, and anti-inflammatory effects (Gul and Hamam, 2005; Mayer and Hamann, 2005; Singh et al., 2005; Prasanna et al., 2010; Harnedy and FitzGerald, 2011). Several scientific studies have demonstrated that cyanobacteria are emerging source for drug discovery (Patterson et al., 1994; Khairy and El-Kassas, 2010; Kumar et al., 2010). Recently, Lee et al. (2014) have reported that multifunctional photo-luminescent green carbon nanotags (G-Tags) synthesized from harmful cyanobacteria has been used as a drug delivery system in cancer therapy. Some fresh water filamentous cyanobacterial strains are also considered as important sources of anticancer biomolecules (Srivastava et al., 2015).

Among different Nostocales species, *Nostoc* sp. is known to produce more than 100 different natural products with antifungal, antimicrobial, antimalarial, and anticancer activities (Shanab et al., 2012). Cryptophycin1 isolated from cyanobacterium *Nostoc* sp. GSV224 has shown to exhibit potent anticancer activity against Adriamycin resistant M17 breast cancer and DMS 273 lung cancer cell lines, KB human nasopharyngeal cancer cells, and LoVo human colorectal cancer cells (Patterson et al., 1991). Several chemically synthesized and naturally occurring analogs of cryptophycin are under second generation clinical trials as potential future anticancer drugs (Liang et al., 2005). Cyanobacterium *Nostoc* sp. is also known to produce bioactive secondary metabolites having antiviral and antitumor activities (Dembitsky and Rezanka, 2005). Nostodione A is a potent antimitotic compound first time isolated from *Nostoc commune* (Kobayashi et al., 1994). Some of the marine cyanobacteria belonging to this particular *Nostoc* genera are *Nostoc linckia, N spongiaeforme var. tenue*, and *Nostoc* sp. ATCC 53789 and GSV 224 are well-recognized sources of potent cytotoxic compounds working against human tumor cell lines (Burja et al., 2001).

In our previous study, we have isolated and characterized novel bioactive compound i.e. Ethyliminomethyl-12-(morpholin-4-ylmethoxy)-5,8,13,16-tetraaza-hexacene-2,3-dicarboxylic acid (EMTAHDCA) from cyanobacterium *Nostoc* sp. MGL001 and proved its application as an antibacterial agent against multidrug resistant gram negative bacteria. The compound has been observed to produce maximum zone of inhibition at 150 μg/mL concentration (Niveshika et al., 2016). In this study, we intended to evaluate the potential application of EMTAHDCA as an anticancer drug resource apart from antibacterial activity through *in silico, in vivo*, and *in vitro* studies.

## Materials and methods

### *In Silico* study

Structural elucidation of EMTAHDCA was performed, as described previously (Niveshika et al., 2016). Three dimensional crystal structures of 11 cancer target receptors with PDBID of 1BIX (Gorman et al., 1997), 1NOW (Mark et al., 2003), 1TE6 (Chai et al., 2004), 2RCW (Madej et al., 2014), 2UVL (Herman et al., 2009), 2VCJ (Brough et al., 2008), 3CRY (Oakley et al., 2010), 3HQU (Chowdhury et al., 2009), 3NMQ (Yun et al., 2011), 5P21 (Pai et al., 1990), and 4B7P (Fogliatto et al., 2013) were retrieved from the protein databank (PDB) (www.rcsb.org/pdb; Table S1). Discovery Studio 3.1 tool was used to optimize 3D models of ligand (EMTAHDCA) as well as 11 cancer related proteins (Gao and Huang, 2011). Preparation of terminus of each proteins were not require to perform. Again discovery studio version 3.1 software was used to remove heteroatom, water molecules, and ligands before docking (Gao and Huang, 2011). MetaPocket 2.0 was used for predicting the active site of a target protein (http://projects.biotec.tu-dresden.de/metapocket/; Huang, 2009; Zhang et al., 2011). An advanced docking tool, Yet Another Scientific Artificial Reality Application (YASARA), was used for docking calculation between receptors and ligand (Krieger and Vriend, 2014). The target protein was set in YASARA to run macro file (dock_run.mcr). YASARA structure provides Autodock and VINA tools to dock ligands with proteins (http://www.yasara.org/docking.htm) using a very simple approach (Morris et al., 1998; Trott and Olson, 2010). AMBER force field was used by docking software YASARA for calculation of energy. AMBER force-field parameters were adjusted iteratively so that the damage done to the structures was minimal (Krieger et al., 2004). Energy minimization was done using default parameter of YASARA Autodock Vina tool (http://www.yasara.org/yamber.htm).

### *In Vitro* study

For all experiments, 15–20 weeks old healthy adult mice (AKR strain) were used because these mice are highly susceptible to develop tumor and have a shorter life-span. Under aseptic condition, about 1 × 10^6^ DLA cells were transplanted intraperitoneally in mice. The life-span of DL bearing mice was 20 ± 2 days, however the life-span of healthy mice is about 18 months, as reported by Bharti and Mishra (2008). The success rate of developing cancer using DLA cells is found to be 100% (Goldie and Felix, 1951). Experimental mice were maintained with standard feeding and drinking at the temperature of 25 ± 2°C for 12/12 h light/dark cycle throughout the study period in the animal house facility of the Zoology Department, Institute of Science, Banaras Hindu University, Varanasi, India as per guidelines of the Institutional Animal Ethical Committee (IAEC), Banaras Hindu University (Bharti and Mishra, 2011).

After successful development of the tumor, mice were anesthetized with chloroform for collecting ascites fluids from the peritoneal cavity. The fluid was then centrifuged at 3,000 rpm for 6 min, and cell pellet was taken and re-suspended in 1 mL of 1X Phosphate Buffered Saline (PBS). Next, 100 μL of cell suspension was mixed with 100 μL of 2X trypan blue (Sigma) to check viable cell density using hemocytometer. The formula used to count viable cells is as follows: No. of cells × dilution factor/ volume (0.4 mm^3^ i.e., 4 squares were counted, each with area of 1 mm^2^, and depth of counting chamber of 0.1 mm).

A preliminary study of DLA cell viability under different concentrations of EMTAHDCA (100, 250, 500, 750, and 1,000 ng/mL) were performed. The 1 mg/mL concentration of EMTAHDCA was prepared by dissolving 1 mg of purified EMTAHDCA in 1 mL of 1% DMSO. Different concentrations of EMTAHDCA were prepared separately using 1 mg/mL concentration as stock solution by formula C_1_V_1_ = C_2_V_2_ where C_1_ represent the stock concentration i.e., 1 mg, C_2_ represent the working concentrations i.e., 100, 250, 500, 750, and 1,000 ng, V_1_ represent the required volume of stock concentration to make working concentration in desired volume (V_2_).

Approximately, 1 × 10^6^ cells were seeded per well in 6-well plate using complete growth medium (RPMI+10% FBS), then cells were treated with different concentrations of EMTAHDCA and incubated for 24 h inside the CO_2_ incubator with 5% CO_2_. Cells treated with only medium served as a control group. Cell imaging was done under EVOS FLoid Cell Imaging Station.

Cytotoxicity of EMTAHDCA at various concentrations (100, 250, 500, 750, and 1,000 ng/mL) were assessed by using MTT assay in DLA cells and normal mouse bone marrow cells (BMC). BMC were isolated from femur bones of 15–20 weeks old adult mice. The mice was anesthetized using chloroform for collecting bone marrow. After that with the help of 24-gauge needle, bone marrow was flushed with pre-warmed PBS and single cells suspension was prepared. The cells were suspended in RPMI-1640 medium supplemented with antibiotic solution and 10% FBS. Cells were seeded in culture plates and maintained in 5% CO_2_ at 37°C for 24 h (Prasad and Koch, 2014).

For MTT assay, 1 × 10^6^ cells/mL DLA and BMC (positive control) were seeded per well in 96-well plate and treated with different concentrations (100–1,000 ng/mL) of EMTAHDCA along with the control (only medium) and vehicle control (1% DMSO). After 24 h, supernatant was removed from each well, washed twice with PBS and MTT solution (5 mg ml^−1^ in PBS) with 100 μL of medium was added. After incubation for another 4 h, resultant formazan crystals were dissolved in dimethyl sulfoxide (100 μL), and the absorbance was measured by a microplate reader (Bio-RAD 680, USA) at 590 nm with a reference wavelength of 620 nm (Prasad and Koch, 2014) and IC_50_ value of EMTAHDCA was calculated using GraphPad Prism 7 software. All experiments were performed in quadruplicate, and the relative cell viability (%) was expressed as a percentage relative to control group.

In case of time dependent study, cells were exposed to 250, 500, and 750 ng/mL of EMTAHDCA along with control and vehicle control and incubated for 12 and 24 h, respectively, and the cell viability test was performed as described above.

### *In Vivo* study

Overall survival time and body weight of DL bearing mice after treatment with EMTAHDCA were evaluated to check anti-cancer and cytotoxic effect of EMTAHDCA in DL induced mice.

DL transplanted mice were randomly divided into two groups, DL group (DL) and DL+ EMTAHDCA treated group. EMTAHDCA treated groups were further divided into two subgroups, 5 and 10 mg/kg B.W. groups, and were administered with respective doses at 6th, 12th, and 18th day of DL transplantation. For the survival time analysis, the percent of mean survival time of EMTAHDCA treated mice (T) over mean survival time of the control group (C) mice were calculated followed by the evaluation of antitumor activities of EMTAHDCA by computing the T/C value of respective groups. The DL group mice were taken as control group for survival time analysis. The body weight was measured at day 0, 3, 6, 9, 12, 15, and 18 because an increase in body weight in ascites tumor model represents tumor mass as explained by Sharma and Koch (2016). Briefly, male mice of 22 g B.W. were taken for the experiment and considered as blank. The graph was plotted after subtracting the body weight of DL mice and 5 and 10 mg/kg B.W. of EMTAHDCA treated DL mice with that of the healthy mice. Kaplan–Meier survival curve was plotted and analyzed by GraphPad Prism7 software using log-rank analysis to examine the level of significance, and *p* < 0.05 was obtained by comparing between normal mice represented as control, DL induced mice and EMTAHDCA treated DL mice groups.

### Statistical analysis

Statistical analysis were performed using software SPSS (version 16.0). For each experiment, six adult male mice were taken in each group (*n* = 6). Each experiments were done 4 times independently. Results were expressed as mean ± standard error of mean (SEM). The IC_50_ value of compound was calculated using GraphPad Prism 7 software. Data were analyzed by chi-square test and One-way ANOVA followed by Tukey *post-hoc* test. A *p* < 0.05 was considered as statistically significant.

## Results

### Molecular docking

**Figure 1:**
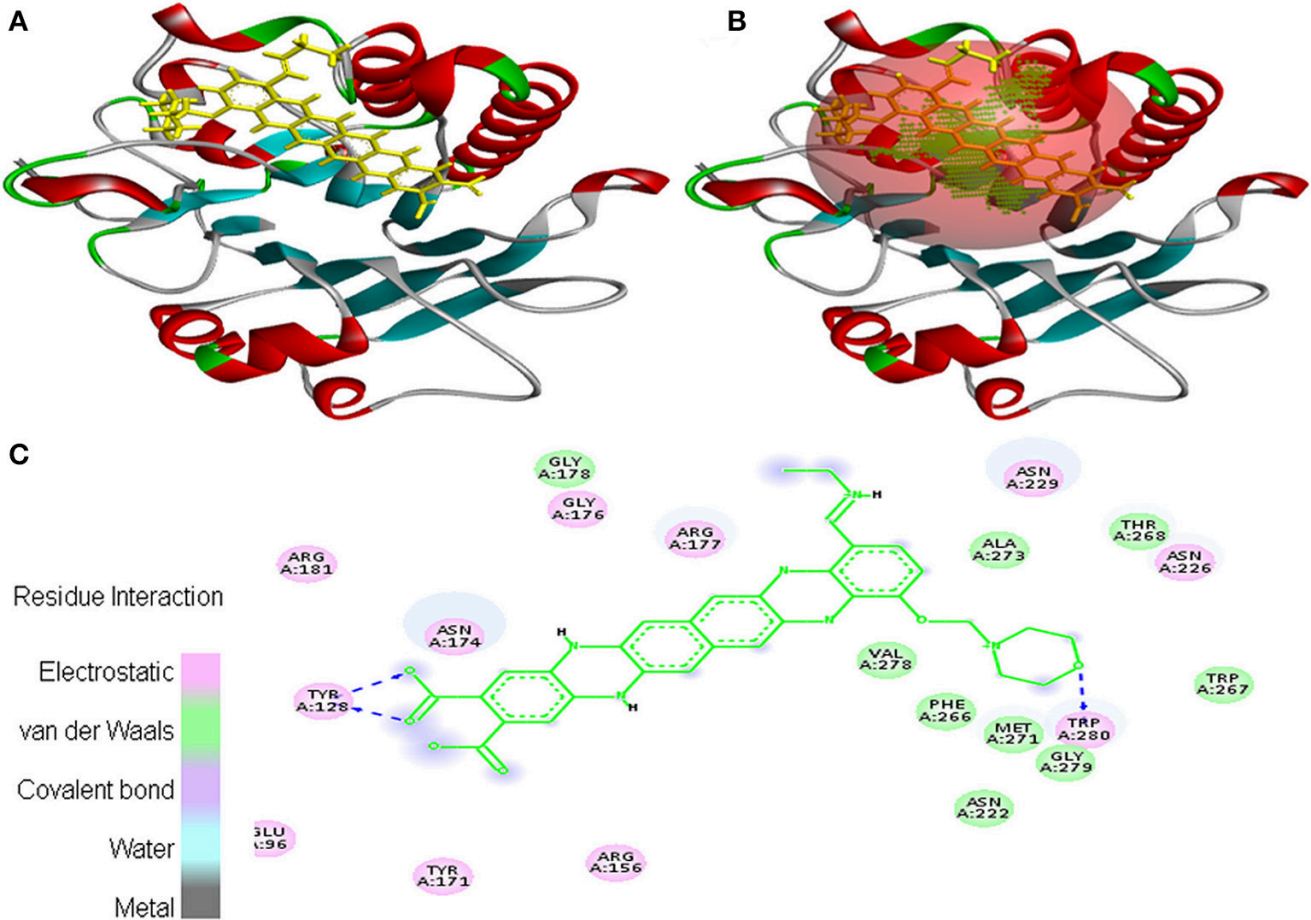
Poses of docked complexes **(A)** *In silico* interaction between selected ligand (EMTAHDCA) and cancer target protein (3CRY), **(B)** Green color in sphere indicates prominent active site where the ligand interacted, **(C)** The 2D level interation clearly mentioned that residues interacted through electrostatic, van der Waals, covalent, and hydrogen bonding.

Molecular docking was successfully performed between the selected ligand (EMTAHDCA) and 11 cancer protein targets (PDB ID: 1BIX, 1NOW, 1TE6, 2RCW, 2UVL, 2VCJ, 3CRY, 3HQU, 3NMQ, 5P21, and 4B7P; Table S1) using YASARA software. The active site of cancer protein targets was determined using Metapocket. Summary of molecular docking results of selected ligand with cancer target protein is represented in Table S2. EMTAHDCA interacted efficiently with the cancer target 3CRY with binding energy of 9.2 and dissociation constant of 169507.6. Contracting receptor residues involved in their interaction through the formation of electrostatic bond, van der Waals force, covalent bond, and hydrogen bond were Gly^23^, Ser^24^, Asn^25^, Arg^30^, Arg^34^, Gly^58^, Lys^59^, Thr^60^, Ser^61^, Gln^62^, Thr^63^, Trp^64^, Ile^68^, Gln^97^, Gln^98^, Met^104^, Tyr^105^, and Tyr^139^ (Figure 1).

**Figure 2:**
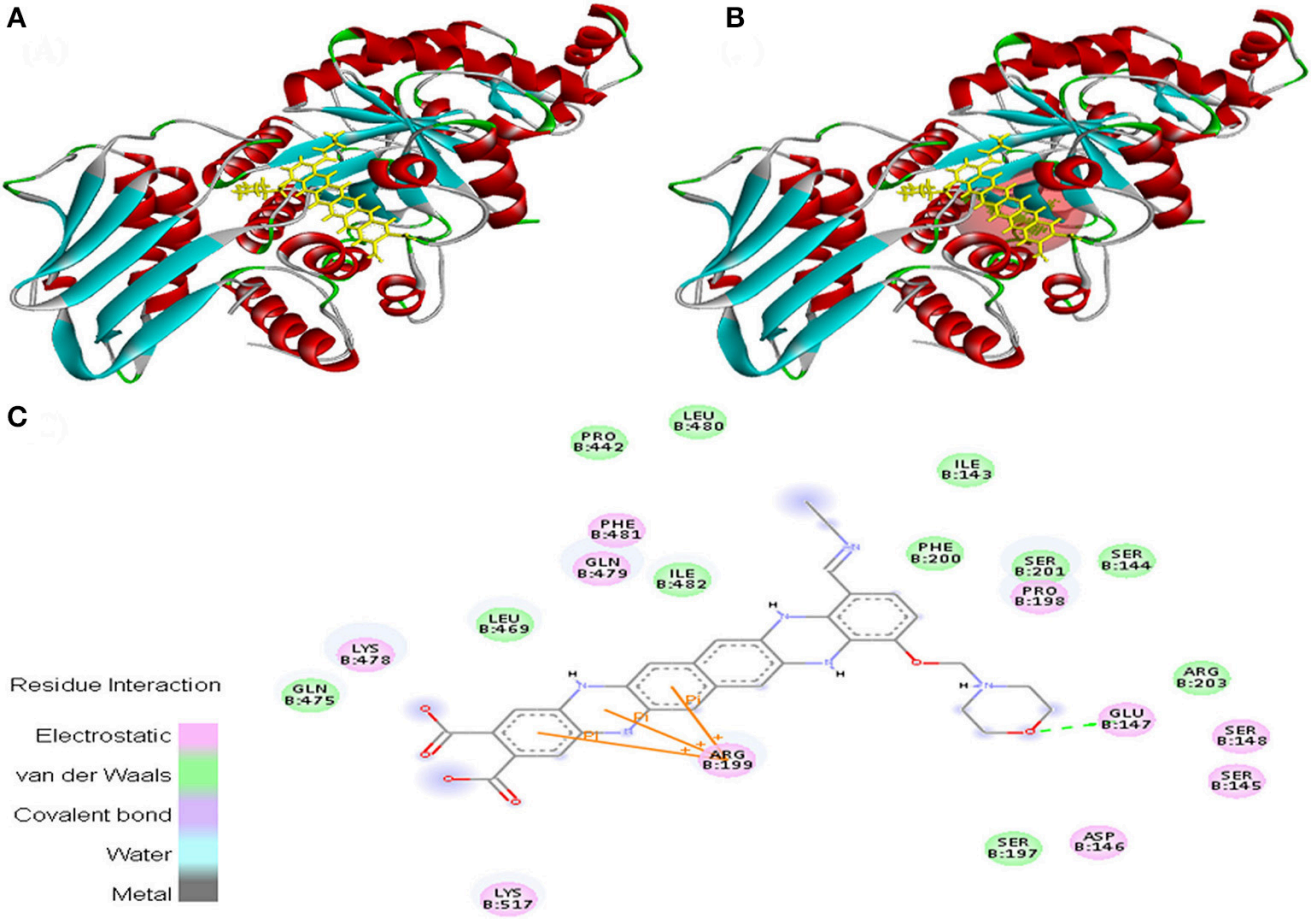
Poses of docked complexes **(A)** *In silico* interaction between selected ligand (EMTAHDCA) and cancer target protein (2VCJ), **(B)** Green color in sphere indicates prominent active site where the ligand interacted, **(C)** The 2D level interation depicted that residues interacted through electrostatic, van der Waals, covalent, and hydrogen bonding.

Moreover, EMTAHDCA efficiently interacted with the cancer target 2VCJ with a good binding energy of 8.5 and less dissociation constant of 567576.2. In this case, active site 1 residues *viz*. Leu^48^, Asn^51^, Ser^52^, Ala^55^, Lys^58^, Tyr^61^, Glu^62^, Asp^93^, Ile^96^, Gly^97^, Met^98^, Asp^102^, Leu^107^, Gly^108^, Phe^138^, Val^150^, Thr^184^, and Val^186^ were found to be involved in the interaction. Residues interacted through electrostatic, van der Waals, covalent, and hydrogen bonding (Figure 2).

**Figure 3:**
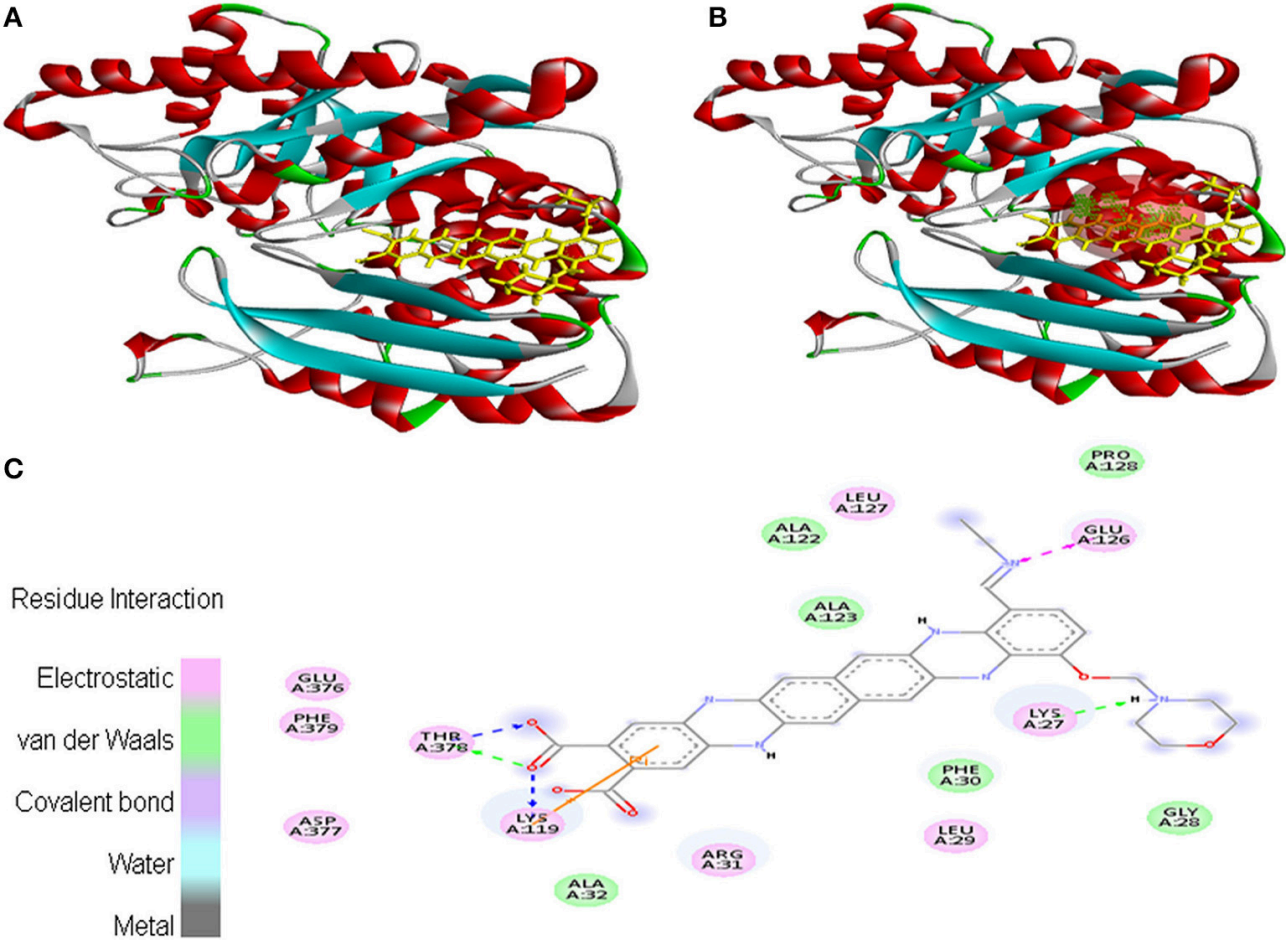
Poses of docked complexes **(A)** *In silico* interaction between selected ligand (EMTAHDCA) and cancer target protein (1TE6) **(B)** Green color in sphere indicates prominent active site where the ligand interacted, **(C)** The 2D level interation indicated that residues interacted through electrostatic, van der Waals, covalent, and hydrogen bonding.

Ligand EMTAHDCA also interacted very efficiently with the cancer protein target 1TE6 with a good binding score of 8.4 and lowest dissociation constant [pM] of 647439.1. Active site 1 residues *viz*. Lys^27^, Gly^28^, Leu^29^, Phe^30^, Arg^31^, Ala^32^, Lys^119^, Ala^122^, Ala^123^, Glu^126^, Leu^127^, Pro^128^, Glu^376^, Asp^377^, Thr^378^, and Phe^379^ were involved in their interaction (Figure 3).

**Figure 4:**
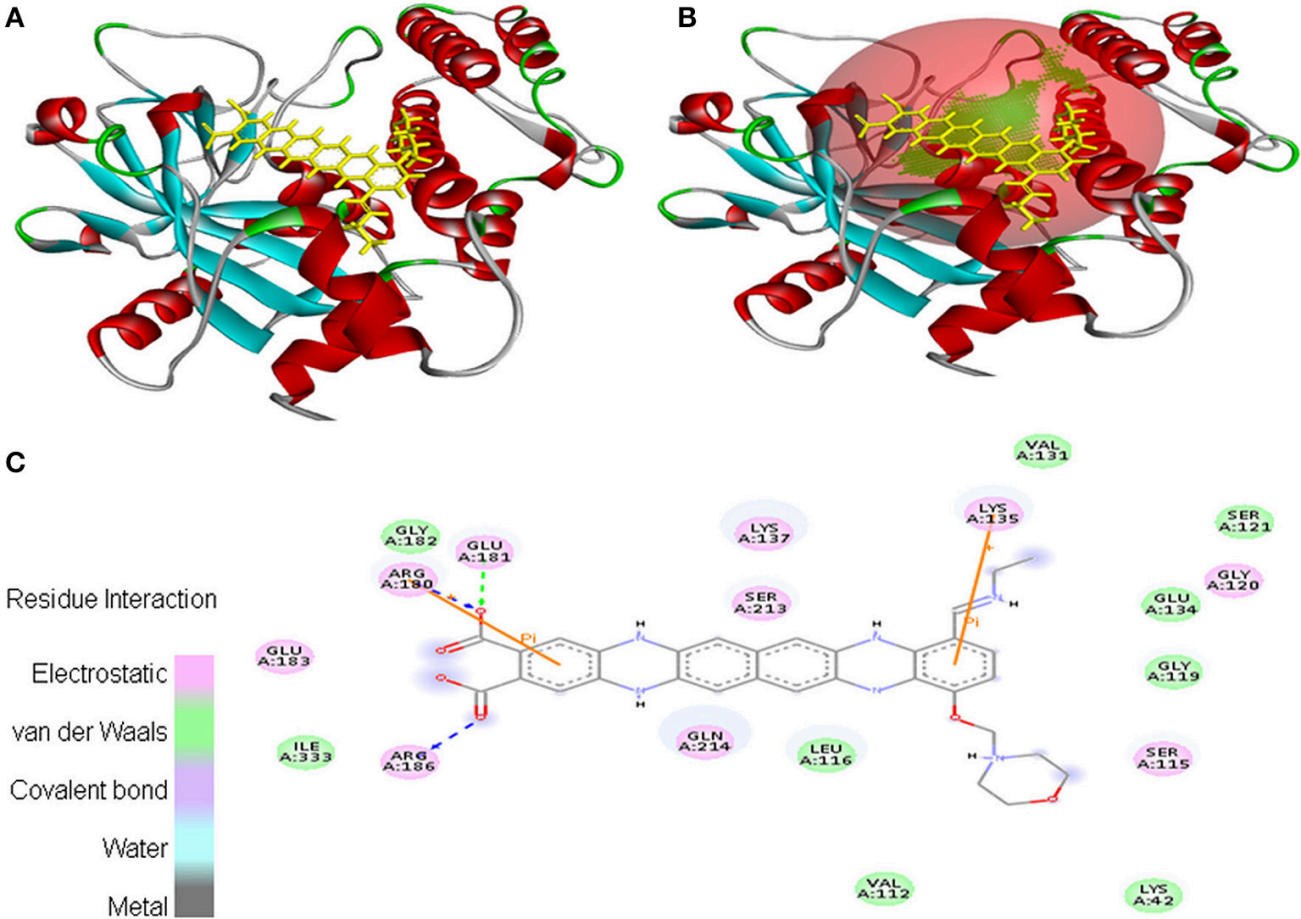
Poses of docked complexes **(A)** *In silico* interaction between selected ligand (EMTAHDCA) and cancer target protein (2RCW), **(B)** Green color in sphere indicates prominent active site where the ligand interacted, **(C)** Residues interacted through electrostatic, van der Waals, covalent, and hydrogen bonding depicted through 2D level interation.

In case of interaction between EMTAHDCA and 2RCW, the binding score was found to be 8.4, whereas the dissociation constant was 689160.3. Amino acid residues involved in this interaction were Lys^42^, Val^112^, Ser^115^, Leu^116^, Gly^119^, Gly^120^, Ser^121^, Val^131^, Glu^134^, Lys^135^, Lys^137^, Arg^180^, Glu^181^, Gly^182^, Glu^183^, Arg^186^, Ser^213^, Gln^214^, and Ile^333^. In addition, 2D view of ligand and protein interaction depicted very clearly that residues interacted through electrostatic, van der Waals, covalent, and hydrogen bonding (Figure 4).

**Figure 5:**
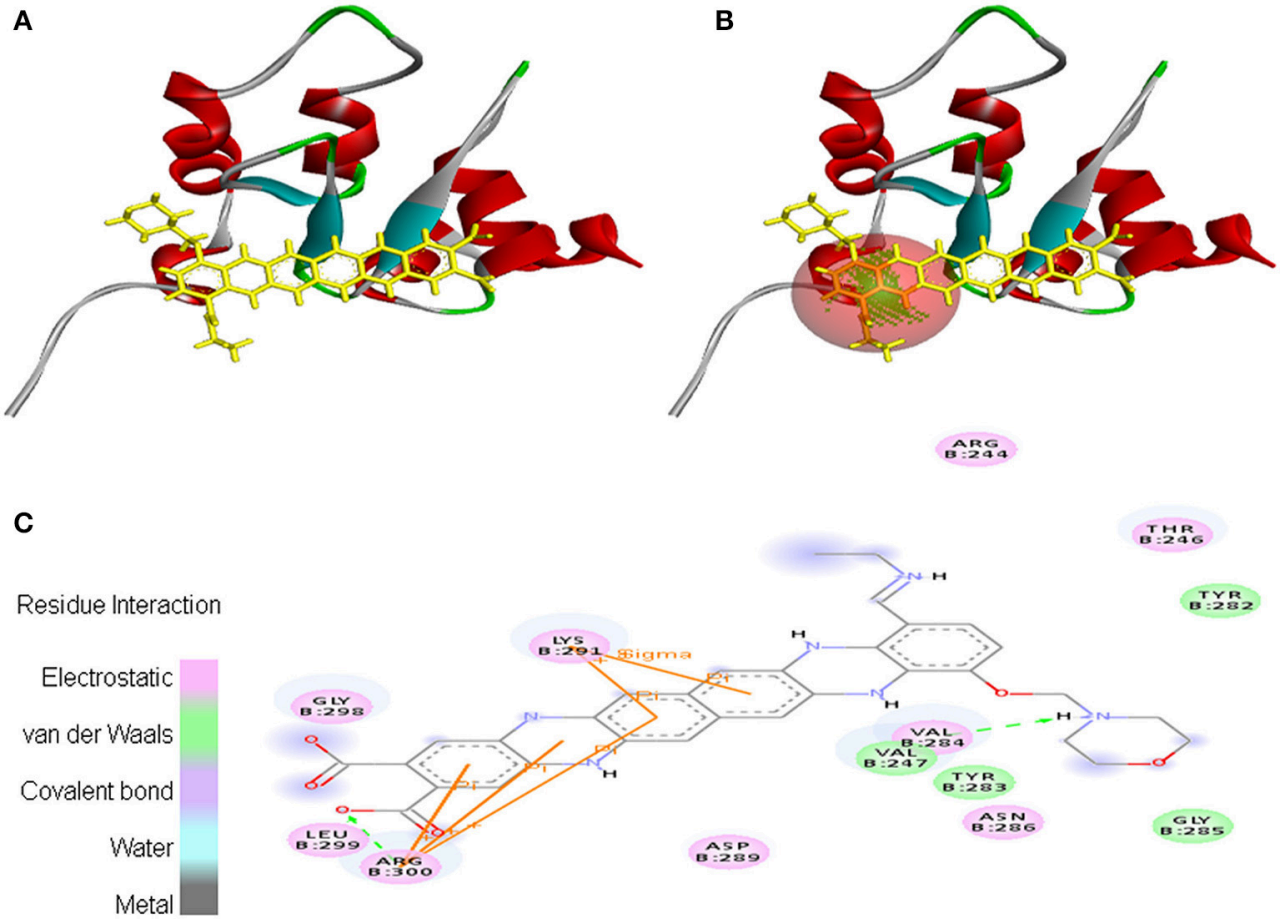
Poses of docked complexes **(A)** *In silico* interaction between selected ligand (EMTAHDCA) and cancer target protein (1BIX), **(B)** Green color in sphere indicates prominent active site where the ligand interacted, **(C)** Amino acid residues interacted through electrostatic, van der Waals, covalent, and hydrogen bonding clearly indicated by their 2D level interation.

Interaction between EMTAHDCA and 1BIX secured binding score of 8.2 and dissociation constant of 1017760.4. Amino acid residues involved in their interaction were Glu^96^, Tyr^128^, Arg^156^, Tyr^171^, Asn^174^, Gly^176^, Arg^177^, Gly^178^, Arg^181^, Asn^222^, Asn^226^, Asn^229^, Phe^266^, Trp^267^, Thr^268^, Met^271^, Ala^273^, Val^278^, Gly^279^, and Trp^280^. These residues were interacted through electrostatic, van der Waals, covalent, and hydrogen bonding (Figure 5).

**Figure 6:**
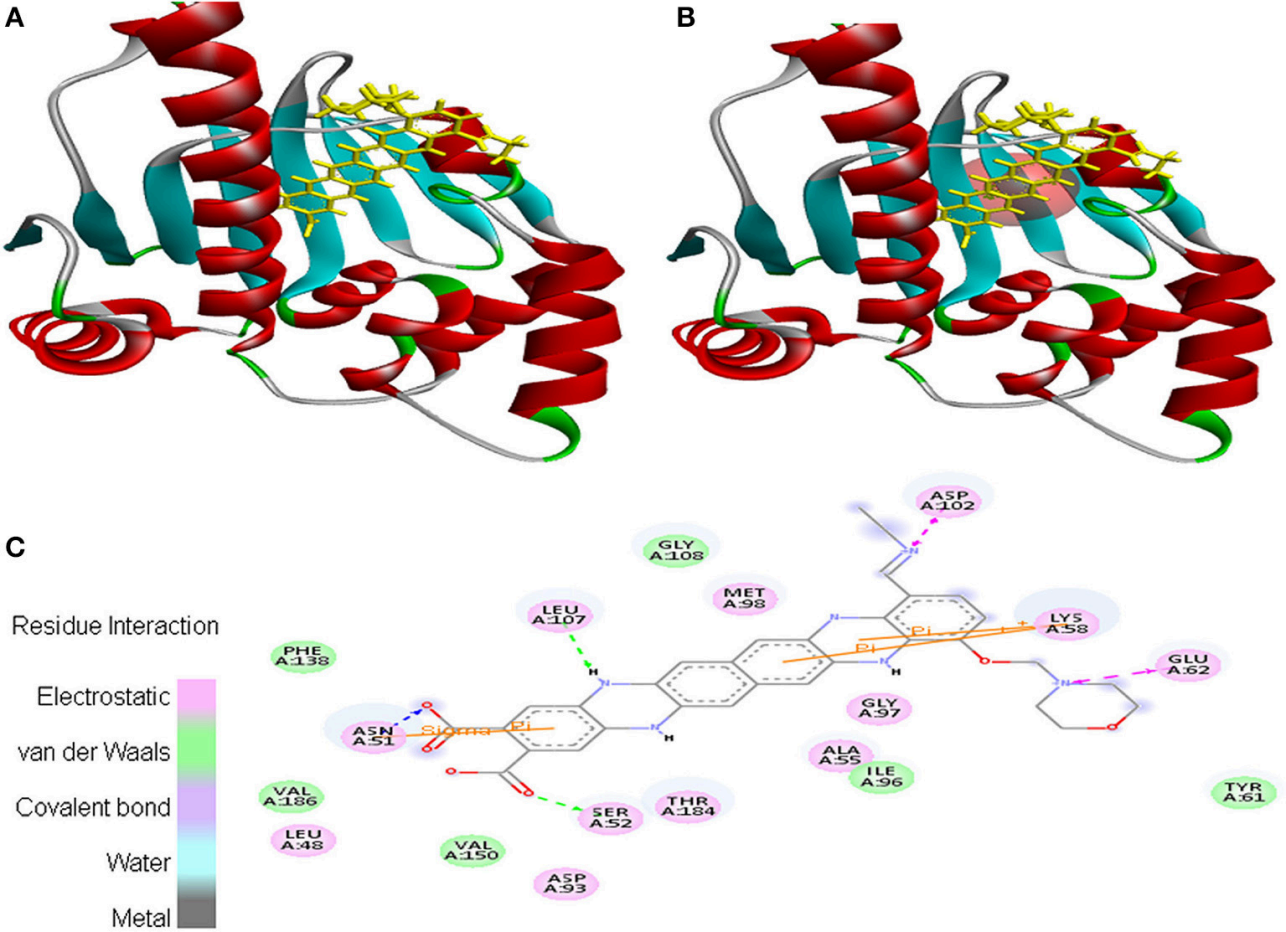
Poses of docked complexes **(A)** *In silico* interaction between selected ligand (EMTAHDCA) and cancer target protein (1NOW), **(B)** Green color in sphere indicates prominent active site where the ligand interacted, **(C)** The 2D level interation of ligand and protein target clearly mentioned that residues interacted through electrostatic, van der Waals, covalent, and hydrogen bonding.

EMTAHDCA interacted with 1NOW at its active site with a high binding score of 8.2 and low dissociation constant of 1024654.8. Active site residues were Ile^143^, Ser^144^, Ser^145^, Asp^146^, Glu^147^, Ser^148^, Ser^197^, Pro^198^, Arg^199^, Phe^200^, Ser^201^, Arg^203^, Pro^442^, Leu^469^, Gln^475^, Lys^478^, Gln^479^, Leu^480^, Phe^481^, Ile^482^, and Lys^517^ (Figure 6).

**Figure 7:**
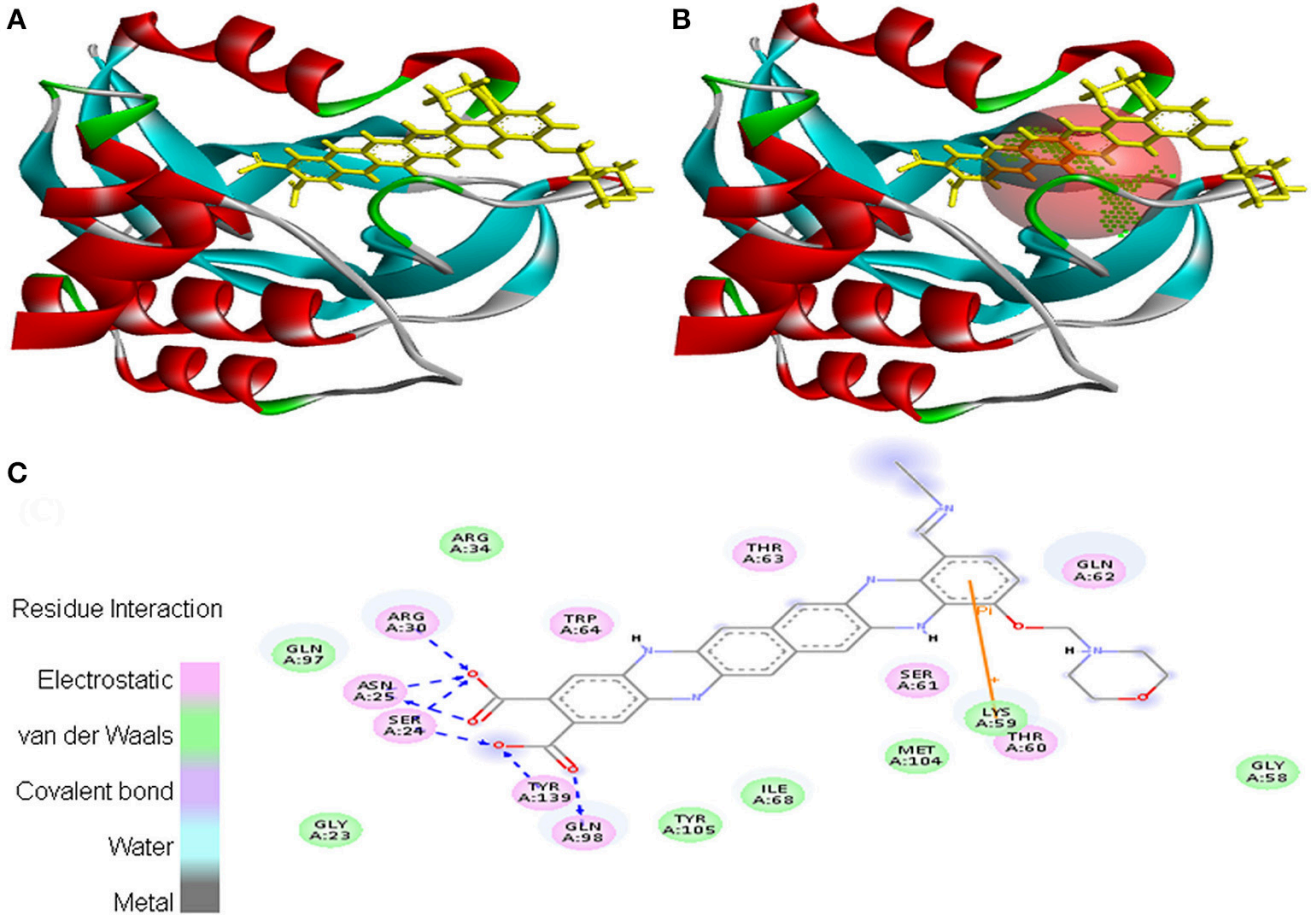
Poses of docked complexes **(A)** *In silico* interaction between selected ligand (EMTAHDCA) and cancer target protein (5P21), **(B)** Green color in sphere indicates prominent active site where the ligand interacted, **(C)** Residues were interacted through electrostatic, van der Waals, covalent, and hydrogen bonding.

In case of EMTAHDCA and 5P21 interaction, binding score was 8.2 and dissociation constant was 1608019.0. Active site 1 residues were Gly^12^, Gly^13^, Val^14^, Gly^15^, Lys^16^, Ser^17^, Ala^18^, Val^29^, Asp^30^, Glu^31^, Tyr^32^, Asp^33^, Pro^34^, Gln^61^, Asn^85^, Asn^86^, Thr^87^, Lys^88^, Lys^117^, and Thr^124^. The 2D view clearly mentioned that residues interacted through electrostatic, van der Waals, covalent, and hydrogen bonding (Figure 7).

**Figure 8:**
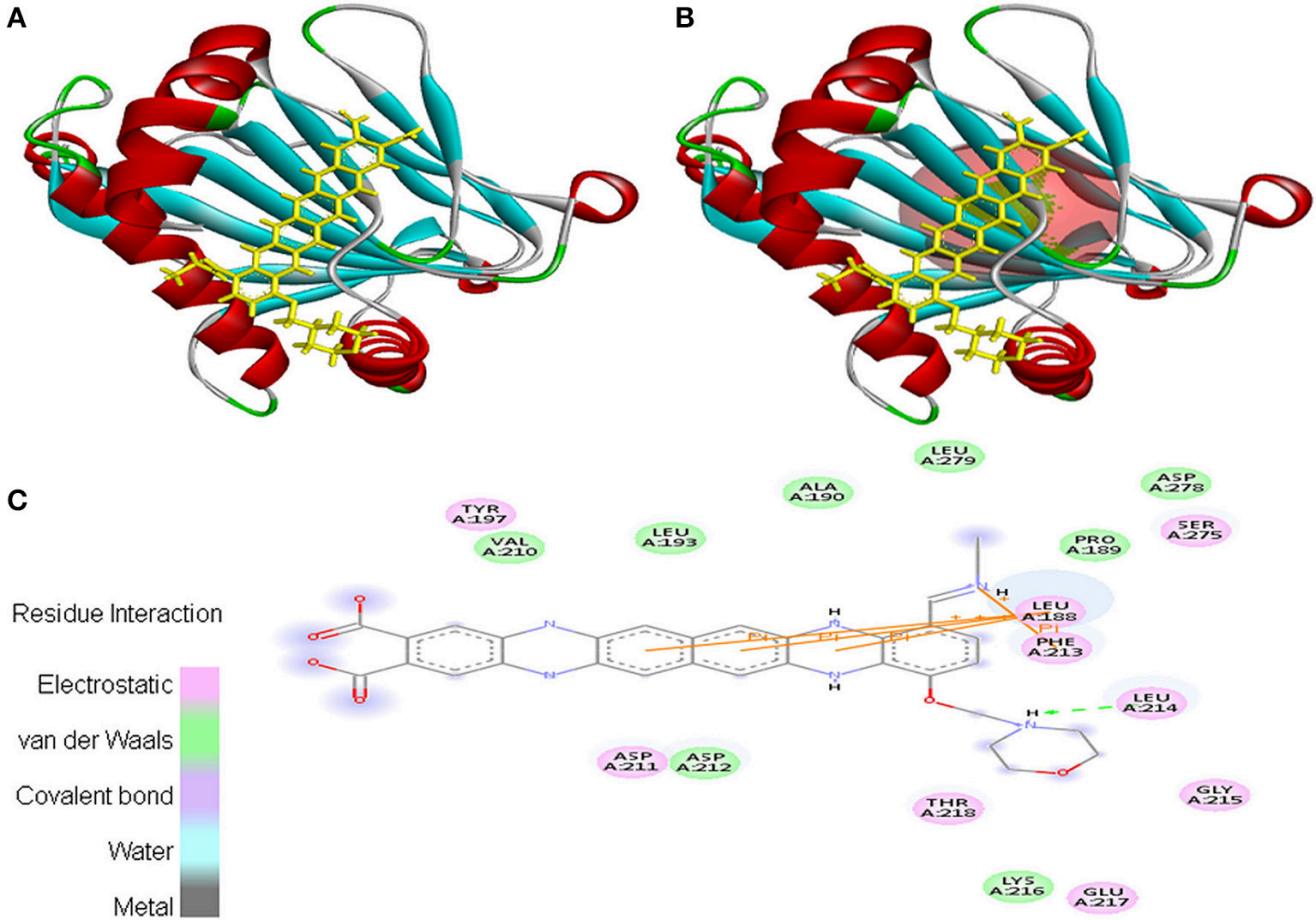
Poses of docked complexes **(A)** *In silico* interaction between selected ligand (EMTAHDCA) and cancer target protein (3NMQ), **(B)** Green color in sphere indicates prominent active site where the ligand interacted, **(C)** 2D level interaction indicated that residues interacted through electrostatic, van der Waals, covalent, and hydrogen bonding.

In case of 3NMQ, EMTAHDCA interacted with a binding score of 7.8 and dissociation constant of 1955781.4. Active site residues *viz*. Asn^51^, Ala^55^, Lys^58^, Ile^96^, Gly^97^, Met^98^,Asp^102^, Leu^107^, Ile^110^, Ser^113^, Gly^114^, Phe^134^, Gly^135^, Val^136^, Phe^138^, Val^150^, His^154^, Thr^184^, and Val^186^ interacted through electrostatic, van der Waals, covalent, and hydrogen bonding (Figure 8).

**Figure 9:**
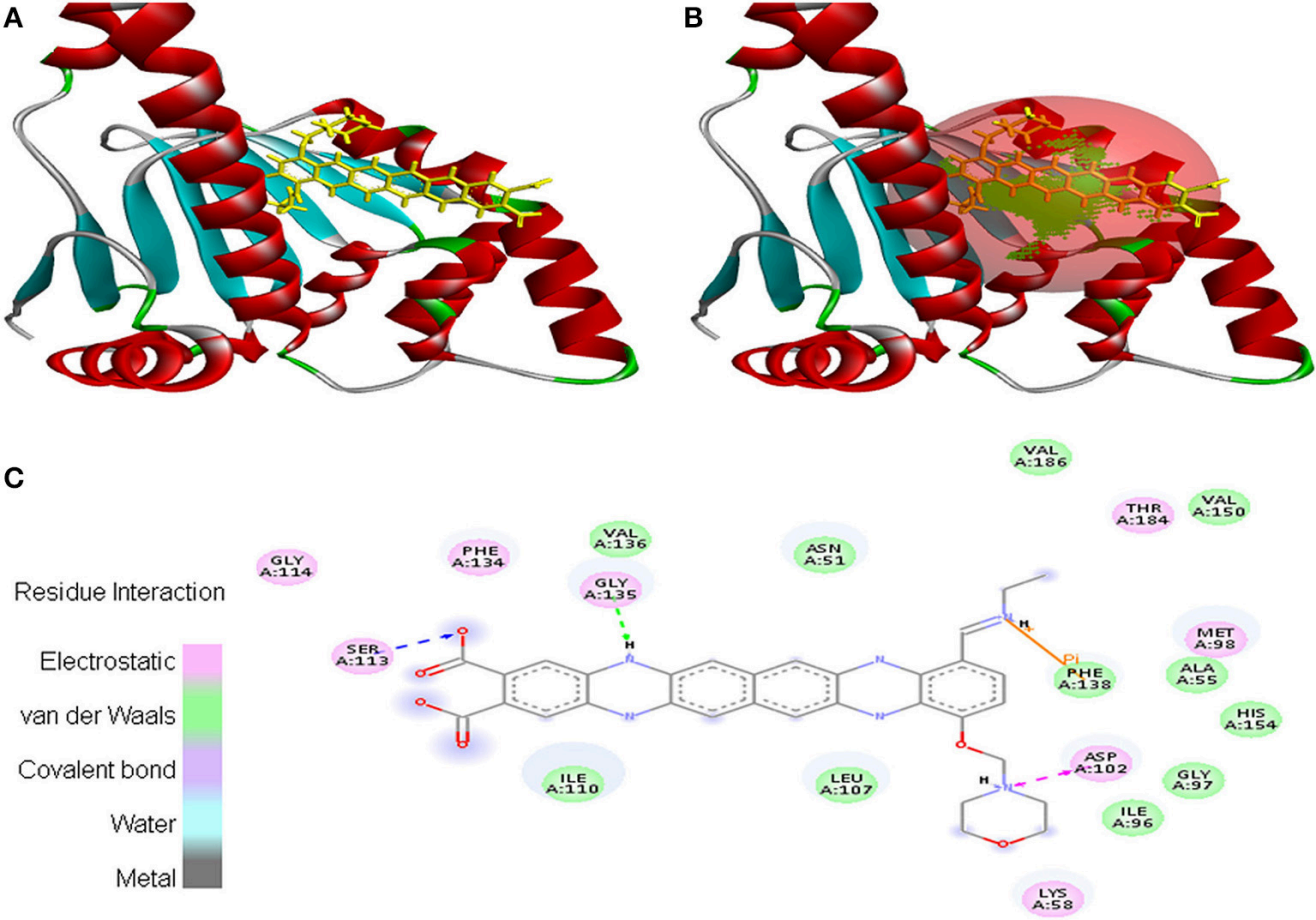
Poses of docked complexes **(A)** *In silico* interaction between selected ligand (EMTAHDCA) and cancer target protein (4B7P), **(B)** Poses of docked complexes, green color in sphere indicates prominent active site where the ligand interacted, **(C)** Residues were interacted through electrostatic, van der Waals, covalent, and hydrogen bonding.

Cancer target protein 4B7P interacted with EMTAHDCA with positive energy of 7.8 and low dissociation constant of 1975688.0. Residues such as Arg^46^, Glu^47^, Ser^50^, Asn^51^, Ser^53^, Asp^54^, Asp^57^, Arg^60^, Lys^112^, Gly^132^, Gln^133^, Gly^135^, Val^136^, Gly^137^, Phe^138^, Phe^213^, Ile^214^, Gly^215^, and Tyr^216^ were involved in this interaction (Figure 9).

**Figure 10:**
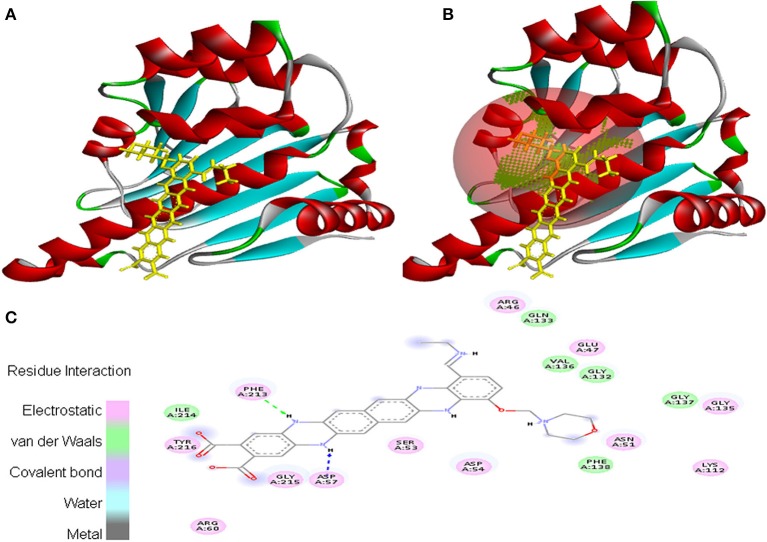
Poses of docked complexes **(A)** *In silico* interaction between selected ligand (EMTAHDCA) and cancer target protein (2UVL), **(B)** Poses of docked complexes, green color in sphere indicates prominent active site where the ligand interacted, **(C)** Amino acid residues interacted through electrostatic, van der Waals, covalent, and hydrogen bonding clearly indicated by their 2D level interation.

Interaction between EMTAHDCA and 2UVL secured a binding score of 7.8 and dissociation constant of 2081812.8. Active site amino acid residues such as Arg^244^, Thr^246^, Val^247^, Tyr^282^, Tyr^283^, Val^284^, Gly^285^, Asn^286^, Asp^289^, Lys^291^, Gly^298^, Leu^299^, and Arg^300^ were involved in their interaction. Electrostatic, van der Waals, covalent, and hydrogen bond were involved in this interaction as clearly mentioned in the 2D view (Figure 10).

**Figure 11:**
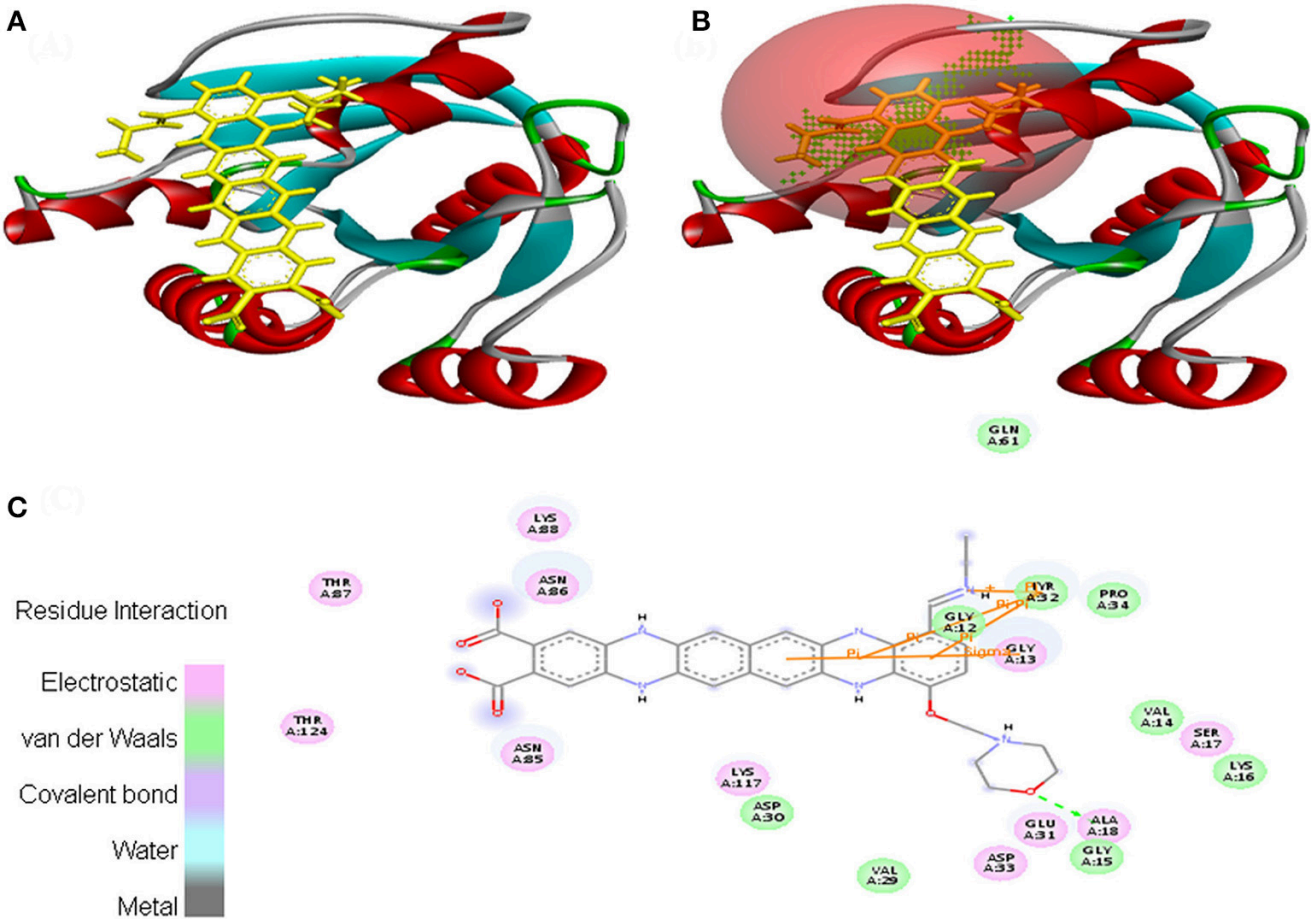
Poses of docked complexes **(A)** *In silico* interaction between selected ligand (EMTAHDCA) and cancer target protein (3HQU), **(B)** Green color in sphere indicates prominent active site where the ligand interacted, **(C)** The 2D level interation depicted that residues interacted through electrostatic, van der Waals, covalent, and hydrogen bonding.

Interaction between EMTAHDCA and 3HQU occurred with positive energy of 7.6 and low dissociation constant of 2645629.8. Amino acid residues such as Leu^188^, Pro^189^, Ala^190^, Leu^193^, Tyr^197^, Val^210^, Asp^211^, Asp^212^, Phe^213^, Leu^214^, Gly^215^, Lys^216^, Glu^217^, Thr^218^, Ser^275^, Asp^278^, and Leu^279^ were involved in their interaction using electrostatic, van der Waals, covalent, and hydrogen bonding (Figure 11).

### *In vitro* and *in vivo* studies

**Figure 12:**
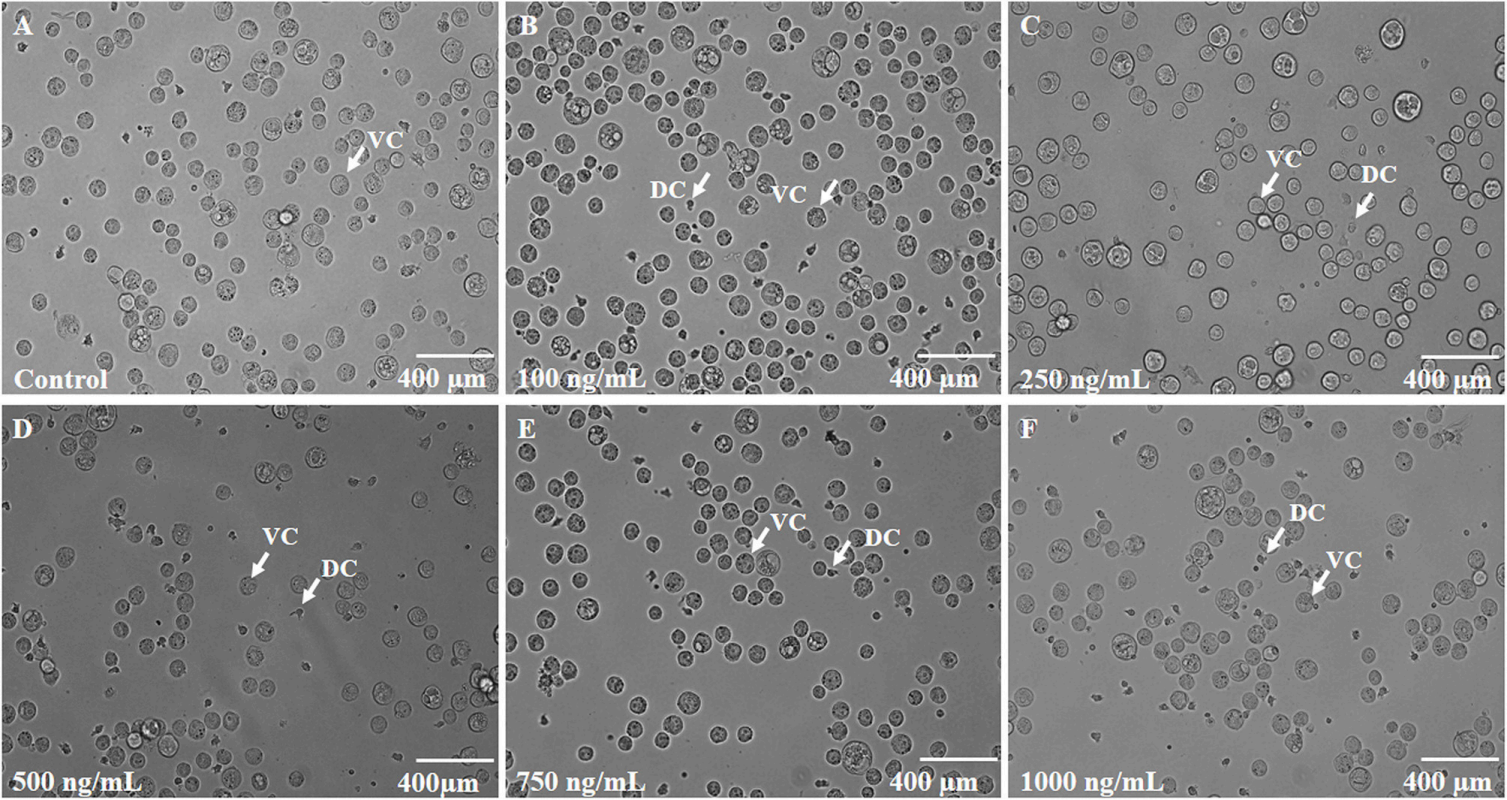
**(A)** Control slide representing DLA cells cultured for 24 h at 37°C with 5% CO_2_ in CO_2_ incubator. **(B–F)** Effect of different concentrations of EMTAHDCA (Control, 100, 250, 500, 750, and 1,000 ng/mL, respectively) on the viability of DLA cells after incubation of 24 h at 37°C with 5% CO_2_ in CO_2_ incubator. DC, Dead cells; VC, Viable cells.

**Figure 13:**
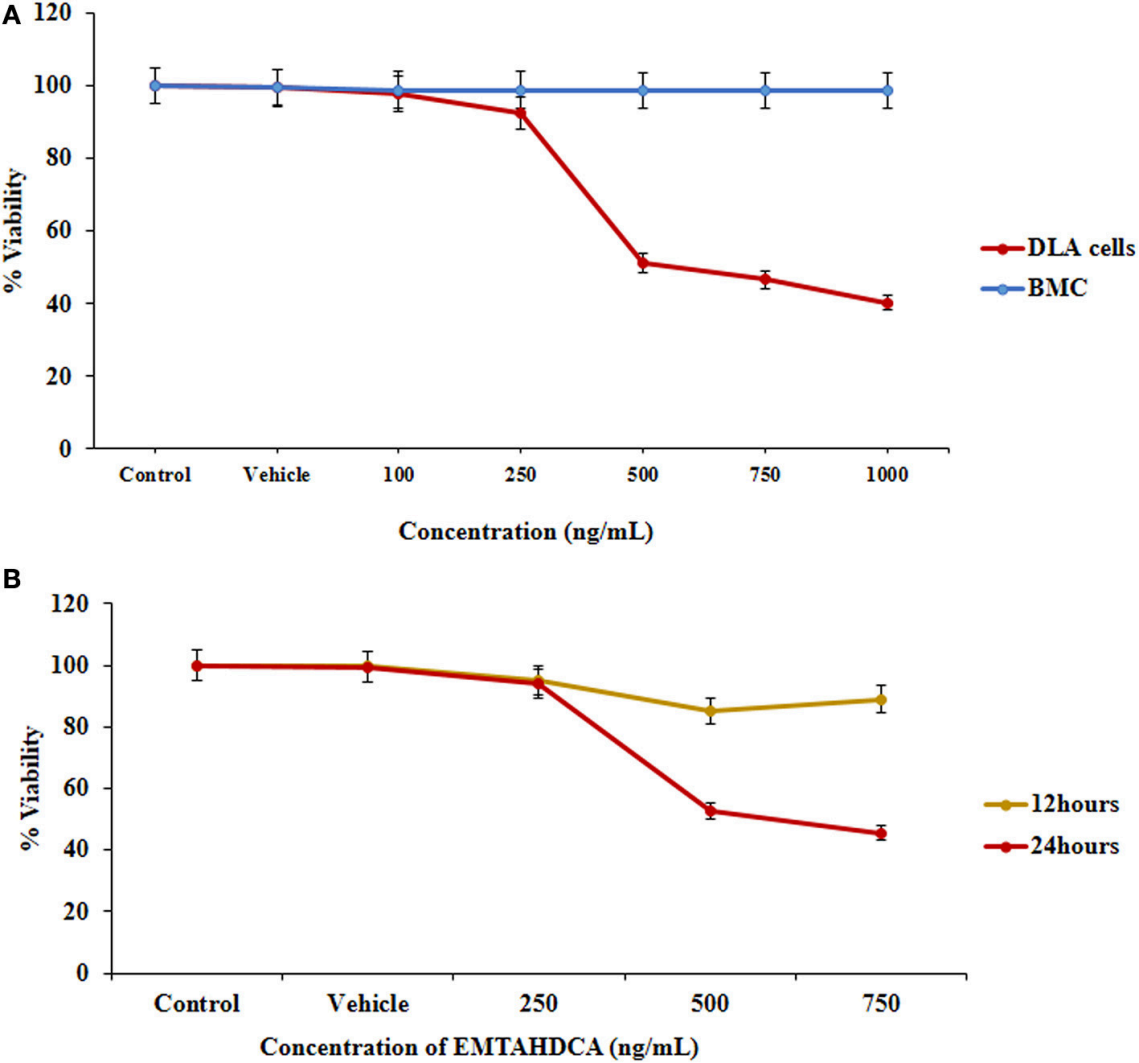
Cell viability was determined by MTT assay, **(A)** Graph represents the cytotoxicity profile of EMTAHDCA against DLA cells and normal mice BMC at different concentrations (100, 250, 500, 750, and 1,000 ng/mL) along with control and vehicle control after 24 h of incubation, **(B)** Effect of EMTAHDCA on the DLA cell viability post 12 and 24 h treatment, respectively.

In order to test the anticancer activity of EMTAHDCA, DLA cells were initially cultured with different concentrations of EMTAHDCA i.e., 100, 250, 500, 750, and 1,000 ng/mL for 24 h (Figures 12A–F) and observed to have cytotoxic effect. Cytotoxic effects of different concentrations of EMTAHDCA on DLA cells and normal BMC were also examined through MTT assay. The percent (%) viability of DLA cells were observed to be 100, 99.8, 97.7, 92.5, 51.2, 46.6, 40.2 at control, vehicle control, 100, 250, 500, 750, and 1,000 ng/mL, respectively whereas percent (%) viability in case of normal BMC were found to be 100, 99.7, 98.9, 98.9, 98.8, 98.7, and 98.7 at control, vehicle control, 100, 250, 500, 750, and 1,000 ng/mL, respectively (Figure 13A, Table S3). The calculated IC_50_ value was found to be 372.4 ng/mL EMTAHDCA against DLA cells. Further, in order to determine time dependent cytotoxicity of EMTAHDCA, MTT assay was performed using 250, 500, and 750 ng/mL concentration of EMTAHDCA at 12 and 24 h time points. The cell viability of DLA cells treated with 500 ng EMTAHDCA was observed 85% after 12 h and 52.5% after 24 h of incubation as compared to the control (100%). However, treatment with 250 ng/mL EMTAHDCA showed 95 and 94% cell viability after 12 and 24 h, respectively. Cell viability of DLA cells treated with 750 ng/mL EMTAHDCA decreased from 89% after 12 h to 45.6% after 24 h (Figure 13B, Table S4).

**Figure 14:**
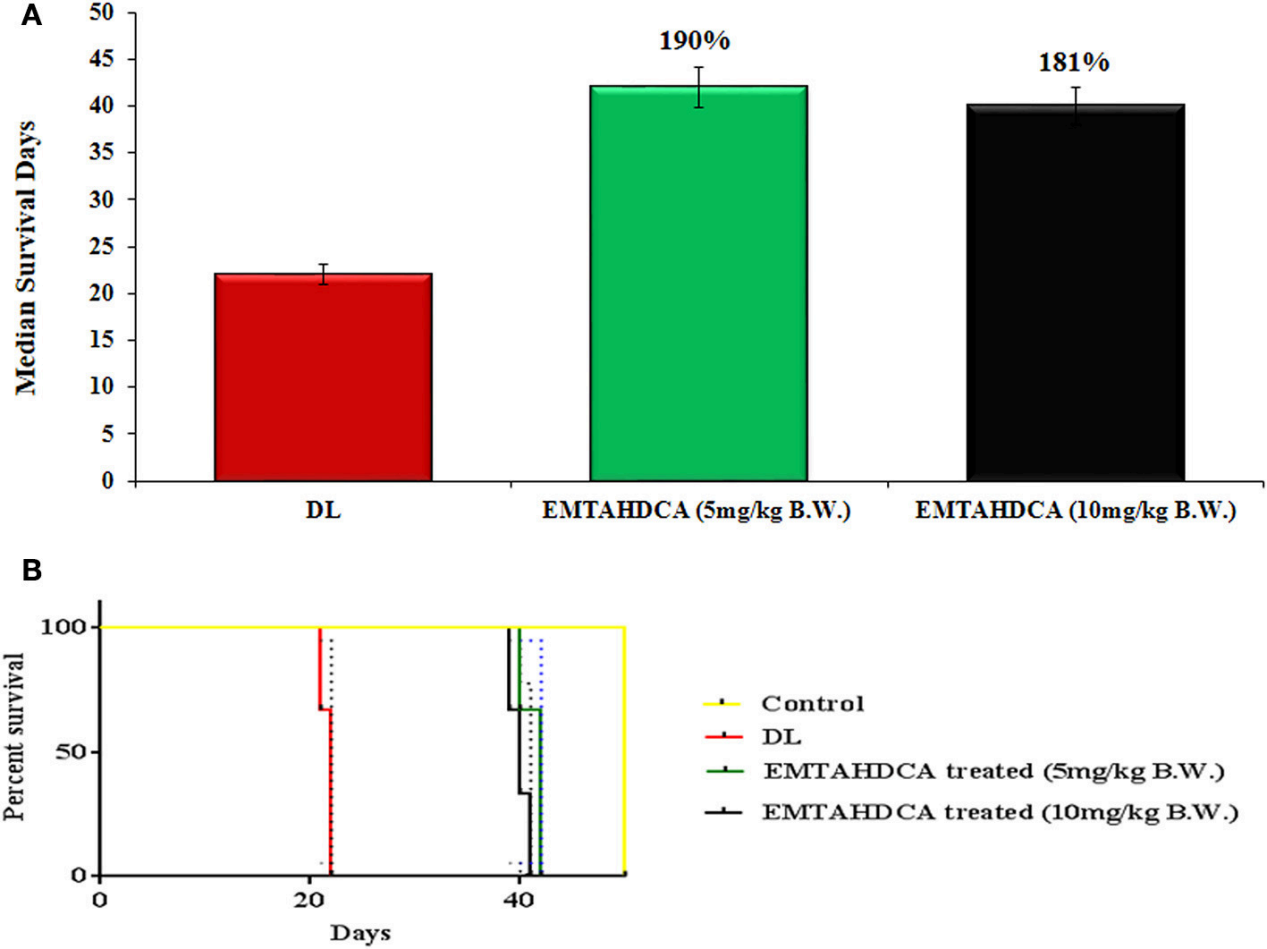
**(A)** Graph represents mean survival/lifespan of DL mice and DL mice treated with EMTAHDCA at two different doses i.e., 5 and 10 mg/kg B.W. The treated/control (T/C) values are shown in percentage (%), **(B)** Kaplan–Meier survival curve for survival of healthy mice (control), DL mice, and DL mice treated with EMTAHDCA at two different doses i.e., 5 and 10 mg/kg B.W. Graph was plotted and analyzed by GraphPad Prism 7 software with log-rank analysis to examine the level of significance and *P* < 0.05 was obtained in between DL and DL treated with EMTAHDCA groups.

**Figure 15:**
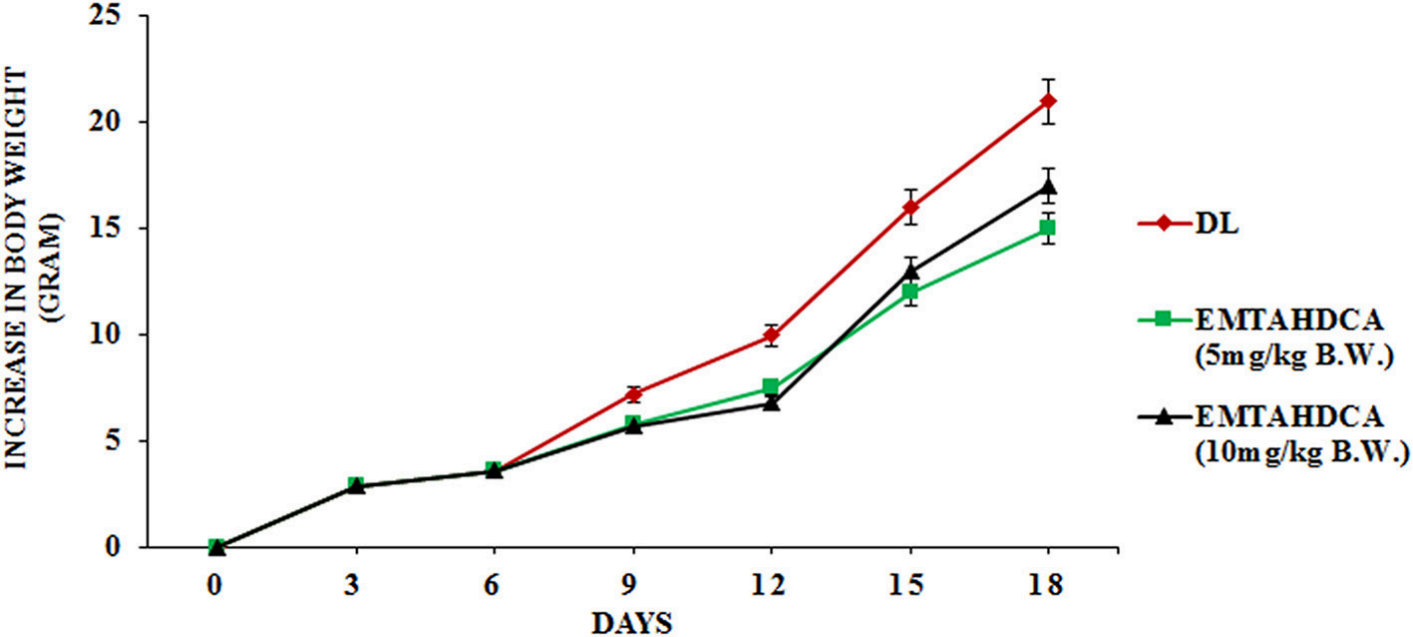
Graph represents effect of two different doses of EMTAHDCA i.e., 5 and 10 mg/kg B.W. on increase in body weight of DL mice from day 0 to 18 after DL transplantation. Values are expressed as mean ± S.E. obtained from 4 independent sets of experiments.

*In vivo* results showed a significant inhibition of tumor growth in mice of EMTAHDCA treated group as compared to the DL group. More importantly, median life span/survival days of tumor bearing mice treated with 5 and 10 mg/kg B.W. of EMTAHDCA significantly increased to 190 and 181%, respectively as compared to DL group (Figure 14A). Increased longevity of EMTAHDCA treated mice as compared to the DL mice was further verified by Kaplan's Meier survival curve using log-rank statistics which showed a significant increase in survival, ~1.9- and 1.81-fold with doses of 5 and 10 mg/kg B.W. of EMTAHDCA respectively as compared to DL mice (Figure 14B). An increase in body weight of DL mice was observed 6 days after the intraperitoneal injection of DLA cells until the death of mice. In contrast, a significant decrease in body weight was observed in DL mice treated with 5 and 10 mg/kg B.W. of the compound on 6th, 12th, and 18th days as compared to DL group (Figure 15). On 9th day, the body weight decreased to 5.8 and 5.7 g after the treatment with 5 and 10 mg/kg B.W. of the compound, respectively, as compared to DL group (7.2 g body weight). On 18th day, the weight decreased to 15 and 17 g after the treatment with 5 and 10 mg/kg B.W. of the compound, respectively, as compared to DL group (21 g body weight). We also found that the administration of EMTAHDCA in healthy mice at a same dose did not cause any major side effects.

## Discussion

Cyanobacteria are known to produce a broad spectrum of metabolites with diverse activities. Since a significant portion of important cyanobacterial drug candidates is still underexplored, it is establishing a new research area of the pharmaceutical science. In our previous study, we reported for the first time that the bioactive compound, EMTAHDCA, from fresh water cyanobacterium *Nostoc* sp. MGL001 has antibacterial activity (Niveshika et al., 2016). Now, this study was designed using *in silico, in vivo*, and *in vitro* approaches to investigate whether this particular natural product also has anticancer effects.

*In silico* docking approach is considered as an emerging field for the rational design of drugs, and therefore, gaining significance in the field of pharmaceutical science (Terstappen and Reggiani, 2001). Here, *in silico* approaches were adopted in order to predict whether this compound has anticancer potential or not. Eleven cancer target proteins were selected (PDBID: 1BIX, 1NOW, 1TE6, 2RCW, 2UVL, 2VCJ, 3CRY, 3HQU, 3NMQ, 5P21, and 4B7P) based on their affiliation with different cancer diseases such as breast cancer, brain tumor, ovarian cancer, colorectal cancer, skin cancer, liver cancer, lung cancer, colon cancer, and bladder cancer (Table S1). Prominent active sites of selected cancer protein targets, which are generally used by commercially available anticancer drugs for binding, have already been reported (Gorman et al., 1997; Mark et al., 2003; Brough et al., 2008; Chowdhury et al., 2009; Herman et al., 2009; Oakley et al., 2010; Yun et al., 2011; Fogliatto et al., 2013; Madej et al., 2014).

In this study, comparative molecular docking calculation indicate that selected ligand (EMTAHDCA) interacts with good binding affinities and best positive energies with all the 11 cancer target proteins at their prominent active sites (predicted by Metapocket) that are generally used by commercially available anticancer drugs for binding. More positive energies indicate stronger binding, and negative energies mean no binding (Morris et al., 1998). Therefore, with the help of advanced molecular docking tools, it was predicted that EMTAHDCA has the ability to work as an anticancer drug agent.

Prediction of anticancer drug potential of EMTAHDCA through *in silico* approaches was further validated using *in vitro* and *in vivo* studies. For the *in vitro* study, DLA cells were selected because of their similarities with some lymphoma and leukemia of human origin (Kumar and Singh, 1996). DLA cells can be maintained by serial transplantation of ascites cells in the intraperitoneal cavity of one mouse to other mouse. To check cytotoxic effect of EMTAHDCA on DLA cells, MTT assay was performed. MTT is one of the reliable and sensitive colorimetric techniques, which is generally used for measuring *in vitro* cytotoxic effects of drugs on cancer cell lines and assessing the viability and proliferation of cancer cells (Meerloo et al., 2011). Five different doses of EMTAHDCA were selected to find out the most effective dose at a minimum concentration. Therefore, we started with the concentration ranging from 100 to 1,000 ng/mL for *in vitro* studies. Cytotoxic effects of EMTAHDCA observed using MTT assay on DLA cells indicate dose and time dependent activities. Our results on *in vitro* studies indicate that treatment with EMTAHDCA cause death of cancerous DLA cells and could act as cytotoxic agent in concentration and time dependent manner with an IC_50_ value at 372.4 ng/mL concentration against DLA cells without affecting normal bone marrow cells even with higher dose of 1,000 ng/mL EMTAHDCA. Since, we do not observed any cytotoxic effect of EMTAHDCA on bone marrow cells in concentration dependent study for 24 h, therefore, we have not taken positive control i.e., bone marrow cells for time dependent study.

*In vivo* studies showed significant increase in median lifespan/survival days of 5 mg/kg B.W. EMTAHDCA-treated mice and decrease in body weight as compared to 10 mg/kg B.W. EMTAHDCA treated and DL mice. However, the drug did not show any significant adverse side-effect on normal mice. The decrease in body weight and increased survival of EMTAHDCA treated mice are in agreement with earlier reports (Ajith and Janardhanan, 2003; Koiri et al., 2009; Singh and Trigun, 2013; Sharma and Koch, 2016) and could be due to regression of tumor growth in the presence of EMTAHDCA as compared to DL mice.

On the whole, our findings suggest that the selected compound EMTAHDCA has potential anticancer properties.

## Conclusion

Our findings based on *in silico, in vitro*, and *in vivo* studies prove that bioactive compound EMTAHDCA isolated from fresh water cyanobacterium *Nostoc* sp. MGL001 has anticancer potential. Therefore, it is worth mentioning that EMTAHDCA can serve as an anticancer drug resource.

## Author contributions

N and AM: designed the experiments; N and SM: performed the experiments; N, EV, AM, SM, and RM: analyzed the data; N and SM: wrote the manuscript and AM critically reviewed the paper.

### Conflict of interest statement

The authors declare that the research was conducted in the absence of any commercial or financial relationships that could be construed as a potential conflict of interest.
