# Supplementary material for: The Combined Use of in Silico, in Vitro, and in Vivo Analyses to Assess Anti-cancerous Potential of a Bioactive Compound from Cyanobacterium Nostoc sp. MGL001
Source: Front Pharmacol. 2017 Nov 27;8:873. doi: 10.3389/fphar.2017.00873 (PMC5711831; doi:10.3389/fphar.2017.00873)
Supplement: Supplementary file 1 [file Table1.DOCX]

**Table S1. List of different cancer protein targets selected for docking with their PDB IDs.**

____________________________________________________________________________________________________________

**Disease Name Target Proteins Specific Type PDB ID Activity References**

____________________________________________________________________________________________________________

Breast Cancer AP endonuclease APE-1 1BIX DNA repair protein Gorman et al. 1997

Breast cancer Breast cancer type 2 BRCA2 1NOW DNA repair protein Mark et al. 2003

susceptibility protein

Brain Tumor Enolase isozymes NSE; HEL-S-279 1TE6 Neuron generation Chai et al. 2004

Ovarian cancer Poly ADP ribose PARP-1 2RCW Programmed cell death Madej et al. 2014

polymerase 1

Colorectal cancer Baculoviral IAP repeat CIAP2 2UVL Inhibitor of apoptosis Herman et al. 2009

containing protein 3

Skin cancer 4, 5 diarylisoxazole HSP90 2VCJ Protein folding and Brough et al. 2008

hsp 90 stability

Liver cancer Gamma-Glutamylcyclo DESC 3CRY Release of Oakley et al. 2010

transferase from  cytochrome c

mitochondria

Lung cancer Hypoxia inducing HIF-1 3HQU Transcription factor Chowdhury et al. 2009

factor 1

Colon cancer Heat shock protein 90 HSP90 3NMQ Protein folding Yun et al. 2011

and stability

Bladder cancer Ongogene protein P21 H-ras 5P21 Growth promoting Pai et al.1990

signal transduction

process

Ovarian cancer Heat shock protein 90 HSP90 4B7P Protein folding Fogliatto et al. 2013

and stability

____________________________________________________________________________________________________________
