# Supplementary material for: The Combined Use of in Silico, in Vitro, and in Vivo Analyses to Assess Anti-cancerous Potential of a Bioactive Compound from Cyanobacterium Nostoc sp. MGL001
Source: Front Pharmacol. 2017 Nov 27;8:873. doi: 10.3389/fphar.2017.00873 (PMC5711831; doi:10.3389/fphar.2017.00873)
Supplement: Supplementary file 2 [file Table2.DOCX]

**Table S2. Summary of molecular docking results of selected ligand (EMTAHDCA) with cancer target proteins.**

| **S.No.** | **Ligand** | **Target** | **PDB ID** | **Binding energy**  **[kcal/mol]** | **Dissociation**  **constant**  **[pM]** | **Contacting receptor**  **residues** |
| --- | --- | --- | --- | --- | --- | --- |
| 1. | EMTAHDCA | Cancer protein | 3CRY | 9.2 | 169507.6 | Gly^23^, Ser^24^, Asn^25^, Arg^30^, Arg^34^, Gly^58^, Lys^59^, Thr^60^, Ser^61^, Gln^62^, Thr^63^, Trp^64^, Ile^68^, Gln^97^, Gln^98^, Met^104^, Tyr^105^, Tyr^139^ |
| 2. | EMTAHDCA | Cancer protein | 2VCJ | 8.5 | 567576.2 | Leu^48^, Asn^51^, Ser^52^, Ala^55^, Lys^58^, Tyr^61^, Glu^62^, Asp^93^, Ile^96^, Gly^97^, Met^98^, Asp^102^, Leu^107^, Gly^108^, Phe^138^, Val^150^, Thr^184^, Val^186^ |
| 3. | EMTAHDCA | Cancer protein | 1TE6 | 8.4 | 647439.2 | Lys^27^, Gly^28^, Leu^29^, Phe^30^, Arg^31^, Ala^32^, Lys^119^, Ala^122^, Ala^123^, Glu^126^, Leu^127^, Pro^128^, Glu^376^, Asp^377^, Thr^378^, Phe^379^ |
| 4. | EMTAHDCA | Cancer protein | 2RCW | 8.4 | 689160.4 | Lys^42^, Val^112^, Ser^115^, Leu^116^, Gly^119^, Gly^120^, Ser^121^, Val^131^, Glu^134^, Lys^135^, Lys^137^, Arg^180^, Glu^181^, Gly^182^, Glu^183^, Arg^186^, Ser^213^, Gln^214^, Ile^333^ |
| 5. | EMTAHDCA | Cancer protein | 1BIX | 8.2 | 1017760.4 | Glu^96^, Tyr^128^, Arg^156^, Tyr^171^, Asn^174^, Gly^176^, Arg^177^, Gly^178^, Arg^181^, Asn^222^, Asn^226^, Asn^229^, Phe^266^, Trp^267^, Thr^268^, Met^271^, Ala^273^, Val^278^, Gly^279^, Trp^280^ |
| 6. | EMTAHDCA | Cancer protein | 1NOW | 8.2 | 1024654.8 | Ile^143^, Ser^144^, Ser^145^, Asp^146^, Glu^147^, Ser^148^, Ser^197^, Pro^198^, Arg^199^, Phe^200^, Ser^201^, Arg^203^, Pro^442^, Leu^469^, Gln^475^, Lys^478^, Gln^479^, Leu^480^, Phe^481^, Ile^482^, Lys^517^ |
| 7. | EMTAHDCA | Cancer protein | 5P21 | 7.9 | 1608019.0 | Gly^12^, Gly^13^, Val^14^, Gly^15^, Lys^16^, Ser^17^, Ala^18^, Val^29^, Asp^30^, Glu^31^, Tyr^32^, Asp^33^, Pro^34^, Gln^61^, Asn^85^, Asn^86^, Thr^87^, Lys^88^, Lys^117^, Thr^124^ |
| 8. | EMTAHDCA | Cancer protein | 3NMQ | 7.8 | 1955781.4 | Asn^51^, Ala^55^, Lys^58^, Ile^96^, Gly^97^, Met^98^, Asp^102^, Leu^107^, Ile^110^, Ser^113^, Gly^114^, Phe^134^, Gly^135^, Val^136^, Phe^138^, Val^150^, His^154^, Thr^184^, Val^186^ |
| 9. | EMTAHDCA | Cancer protein | 4B7P | 7.8 | 1975688.0 | Arg^46^, Glu^47^, Ser^50^, Asn^51^, Ser^53^, Asp^54^, Asp^57^, Arg^60^, Lys^112^, Gly^132^, Gln^133^, Gly^135^, Val^136^, Gly^137^, Phe^138^, Phe^213^, Ile^214^, Gly^215^, Tyr^216^ |
| 10. | EMTAHDCA | Cancer protein | 2UVL | 7.8 | 2081812.8 | Arg^244^, Thr^246^, Val^247^, Tyr ^282^, Tyr^283^, Val^284^, Gly^285^, Asn^286^, Asp^289^, Lys^291^, Gly^298^, Leu^299^, Arg^300^ |
| 11. | EMTAHDCA | Cancer protein | 3HQU | 7.6 | 2645629.8 | Leu^188^, Pro^189^, Ala^190^, Leu^193^, Tyr^197^, Val^210^, Asp^211^, Asp^212^, Phe^213^, Leu^214^, Gly^215^, Lys^216^, Glu^217^, Thr^218^, Ser^275^, Asp^278^, Leu^279^ |
