# Supplementary material for: The Combined Use of in Silico, in Vitro, and in Vivo Analyses to Assess Anti-cancerous Potential of a Bioactive Compound from Cyanobacterium Nostoc sp. MGL001
Source: Front Pharmacol. 2017 Nov 27;8:873. doi: 10.3389/fphar.2017.00873 (PMC5711831; doi:10.3389/fphar.2017.00873)
Supplement: Supplementary file 3 [file Table3.DOCX]

**Table S3. MTT assay showing % viability of BMC and DLA cells treated with different concentrations of EMTAHDCA (100, 250, 500, 750 and 1000 ng/mL along with control and vehicle control) incubated for 24 hours.**

_____________________________________________________________________________________

**Different concentrations % Viability**

**of compound (ng/mL) _________________________________________________**

**BMC DLA cells**

______________________________________________________________________________

Control 100 100

Vehicle control 99.8 99.7

100 98.9 97.7

250 98.9 92.5

500 98.8 51.2

750 98.7 46.6

1000 98.7 40.2 ______________________________________________________________________________
