# Supplementary material for: The Combined Use of in Silico, in Vitro, and in Vivo Analyses to Assess Anti-cancerous Potential of a Bioactive Compound from Cyanobacterium Nostoc sp. MGL001
Source: Front Pharmacol. 2017 Nov 27;8:873. doi: 10.3389/fphar.2017.00873 (PMC5711831; doi:10.3389/fphar.2017.00873)
Supplement: Supplementary file 4 [file Table4.DOCX]

**Table S4. MTT assay showing % viability of DL cells treated with different concentrations of EMTAHDCA i.e. Control, 250, 500 and 750 ng/mL incubated at 12 and 24 hour time points.**

_____________________________________________________________________________________

**Different concentrations 12 hour 24 hour**

**of compound (ng/mL) (%Viability) (%Viability)**

______________________________________________________________________________

Control 100 100

Vehicle control 99.8 99.7

250 95 94

500 85 52.5

750 89 45.6

______________________________________________________________________________
